# Sex-specific positive and negative consequences of avoidance training during childhood on adult active avoidance learning in mice

**DOI:** 10.3389/fnbeh.2013.00143

**Published:** 2013-10-16

**Authors:** Almuth Spröwitz, Jörg Bock, Katharina Braun

**Affiliations:** ^1^Department of Zoology/Developmental Neurobiology, Institute of Biology, Otto von Guericke University MagdeburgMagdeburg, Germany; ^2^Center for Behavioral Brain SciencesMagdeburg, Germany

**Keywords:** active avoidance learning, infant learning, feedback learning, sex differences, ontogeny of learning

## Abstract

In humans and animals cognitive training during childhood plays an important role in shaping neural circuits and thereby determines learning capacity later in life. Using a negative feedback learning paradigm, the two-way active avoidance (TWA) learning, we aimed to investigate in mice (i) the age-dependency of TWA learning, (ii) the consequences of pretraining in childhood on adult learning capacity and (iii) the impact of sex on the learning paradigm in mice. Taken together, we show here for the first time that the beneficial or detrimental outcome of pretraining in childhood depends on the age during which TWA training is encountered, indicating that different, age-dependent long-term “memory traces” might be formed, which are recruited during adult TWA training and thereby either facilitate or impair adult TWA learning. While pretraining during infancy results in learning impairment in adulthood, pretraining in late adolescence improved avoidance learning. The experiments revealed a clear sex difference in the group of late-adolescent mice: female mice showed better avoidance learning during late adolescence compared to males, and the beneficial impact of late-adolescent pretraining on adult learning was more pronounced in females compared to males.

## Introduction

It is a widely accepted concept, mainly based on findings in the sensory systems that early experience affects the development and maturation of CNS function and thereby shapes adult behavioral competence (Shors, 2004; Sullivan et al., 2006; Hunt et al., 2007; Lupien et al., 2009). Furthermore, there is a host of literature providing evidence for specific “critical” developmental time windows, during which the environment has its greatest beneficial or adverse impact (Zaharia et al., 1996; Heim and Nemeroff, 2001; Pollak, 2003; Romeo et al., 2003; Bock et al., 2005; Ruedi-Bettschen et al., 2005).

Using the two-way active avoidance (TWA) learning paradigm we applied the concept of early cognitive and sensory experience to an aversive learning paradigm, which allowed us to test the hypothesis that infant training has a pronounced and long-lasting impact on behavioral and learning strategies in adulthood. Following the concept of “critical” phases in development we predicted that the beneficial and/or detrimental consequences on adult learning critically depends on the maturity of the young animal at the time of its exposure to training. We chose to investigate the TWA paradigm because it is a type of feedback-based learning, which requires the ability to incorporate performance feedback into the learning process. In humans and other animals positive as well as negative feedback (as represented in the TWA paradigm) is important to optimize behavioral strategies, and studies in humans revealed that this ability matures postnatally (van Duijvenvoorde et al., 2008).

The ontogeny of TWA learning has been well studied in rats (McLaughlin et al., 1975; McNamara et al., 1977; Bauer, 1978; Myslivecek and Hassmannova, 1979; Gruss et al., 2010). A previous study (Schäble et al., 2007) in female rats revealed that infants [postnatal day (PND) 17–21; same age as the infant mice in the present study] are not able to develop a successful avoidance strategy. Nevertheless, as adults these animals, as well as animals, which were pretrained during preadolescence and adolescence all showed accelerated avoidance learning in adulthood compared to non-pretrained adults (Gruss et al., 2010). Since mice are becoming increasingly popular also for behavioral studies due to the availability of genetically modified mutants, another aim was to analyse the age-dependency of TWA learning in C57Bl/6 mice, i.e., the first mouse strain whose genome was fully sequenced, and which is most commonly used as a background strain for the generation of congenics with spontaneous or induced mutations. Finally, since the majority of behavioral studies are restricted to the analysis of male individuals, another aim of this study was to compare male and female TWA learning performance.

## Materials and methods

### Animals and rearing conditions

The C57Bl/6 mice were bred in the colony at the Institute of Biology (University Magdeburg, Germany). All animals were housed under standard laboratory conditions (temperature: 22 ± 2°C, humidity: 55 ± 5%) with a 12 h light/12 h dark cycle and *ad libitum* access to food and water. The pups were weaned at PND 21 and were housed together with same-sex siblings. All experiments were in compliance with the European Communities Council Directive (86/609/EEC). The experimental protocol was approved by the ethics committee of the government of the state of Saxony-Anhalt.

### Two-way active avoidance learning

All experiments were conducted in a fully automated shuttle box (TSE Systems, Germany) between 8 a.m. and 2 p.m. during the light phase. The shuttle box consisted of two equal compartments (each 140 × 155 × 160 mm) separated by an opening to allow the mice to move from one compartment to the other.

**Figure 1:**
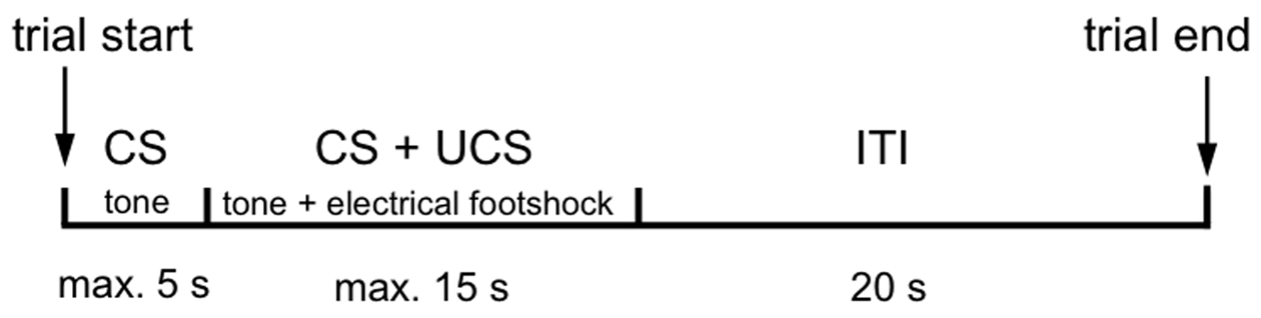
**Schedule of a single trial**.

Each training day started with a 3 min habituation phase allowing the mice to freely explore the environment. The daily training session consisted of 50 trials with the following parameters: A 2.4 kHz tone was applied as conditioned stimulus (CS) for 5 s, followed by a simultaneous application of CS with an unconditioned stimulus (UCS), which was a 0.3 mA footshock with a maximal duration of 15 s. The trials were separated by a 20 s inter-trial interval (ITI). The trial paradigm is shown in Figure 1.

The animals were able to show three responses:

1. Change the compartment during the presentation of the CS prior to the onset of the UCS (=AVOIDANCE)
2. Change the compartment after the UCS onset (=ESCAPE) or
3. No change of the compartment during CS and UCS presentation (=FAILURE).

The following parameters were recorded: number of avoidances, escapes and failures, avoidance and escape latencies and number of compartment changes during the ITI.

### Experimental groups

To study the ontogeny of avoidance learning the animals were assigned to one of the following experimental groups:

#### Infants

Animals were trained as infants (PND 17–21) in the shuttle box paradigm on 5 consecutive days (females: *n* = 12; males *n* = 10).

#### Preadolescents

Animals were trained during preadolescence (PND 24–28) in the shuttle box paradigm on 5 consecutive days (females: *n* = 13; males *n* = 13).

#### Adolescents

Animals were trained during adolescence (PND 31–35) in the shuttle box paradigm on 5 consecutive days (females: *n* = 13; males *n* = 13).

#### Late adolescents

Animals were trained during late adolescence (PND 38–42) in the shuttle box paradigm on 5 consecutive days (females: *n* = 12; males *n* = 11).

To study the age-dependent consequences of TWA pretraining on adult TWA learning the same animals from the ontogenetic study (see above) were retrained in adulthood:

#### Pretrained as infants

Animals were pretrained as infants (PND 17–21) in the shuttle box paradigm on 5 consecutive days and were retrained as adults (PND 80–84) in the same paradigm for 5 consecutive days (females: *n* = 12; males *n* = 10).

#### Pretrained as preadolescents

Animals were pretrained during preadolescence (PND 24–28) in the shuttle box paradigm on 5 consecutive days and were retrained as adults (PND 80–84) in the same paradigm for 5 consecutive days (females: *n* = 13; males *n* = 13).

#### Pretrained as adolescents

Animals were pretrained during adolescence (PND 31–35) in the shuttle box paradigm on 5 consecutive days and were retrained as adults (PND 80–84) in the same paradigm for 5 consecutive days (females: *n* = 13; males *n* = 13).

#### Pretrained as late adolescents

Animals were pretrained during late adolescence (PND 38–42) in the shuttle box paradigm on 5 consecutive days and were retrained as adults (PND 80–84) in the same paradigm for 5 consecutive days (females: *n* = 12; males *n* = 11).

#### Non-pretrained adults

Animals were only trained as adults (PND 80–84) in the shuttle box paradigm for 5 consecutive days (females: *n* = 25; males *n* = 24).

The classification of the age groups was adapted according to Tirelli et al. (2003) and Spear (2004).

### Statistical analysis

For an overall analysis including all parameters a three-way repeated-measures analysis of variance (ANOVA) was applied to analyse the data of the eight (see previous section) pretrained groups. The non-pretrained adult group was not included in this analysis. The three main factors were sex (male and female), training (pretraining and adult retraining) and age during pretraining (infant, preadolescent, adolescent, and late-adolescent). The day of training (day 1–5) was used as repeated-measures factor.

To reveal sex-specific differences in the ontogeny of avoidance learning a two-way repeated-measures ANOVA was applied with age (infant, preadolescent, adolescent, late adolescent, and adult), sex (male and female) as main factors and day of training (day 1–5) as repeated-measures factor.

To reveal sex-specific consequences of TWA pretraining on adult avoidance learning a two-way repeated-measures ANOVA was applied with pretraining condition (pretrained as infants, pretrained as preadolescents, pretrained as adolescents, pretrained as late adolescents and non-pretrained adults) during adult training, sex (male and female) as main factors and day of training (day 1–5) as repeated-measures factor.

To study the ontogeny of avoidance learning for males and females separately, a one-way repeated-measures ANOVA was applied with age (infant, preadolescent, adolescent, late adolescent, and adult) as main factor and day of training (day 1–5) as repeated-measures factor. The statistical analysis was followed by *post-hoc* least significant difference multiple comparison tests (LSD), if applicable. For a detailed analysis of each training day we used the univariate ANOVA followed by LSD *post-hoc* comparisons whenever appropriate.

To investigate the impact of TWA pretraining on adult avoidance learning for males and females separately, a one-way repeated-measures ANOVA was applied with pretraining condition (pretrained as infants, pretrained as preadolescents, pretrained as adolescents, pretrained as late adolescents, and non-pretrained) as main factor and day of training (day 1–5) as repeated-measures factor. The statistical analysis was followed by *post-hoc* least significant difference multiple comparison tests (LSD), if applicable. Additionally, for a detailed analysis of each training day we used the univariate ANOVA followed by LSD *post-hoc* comparisons whenever appropriate.

Data analysis and diagram compilation were performed using SPSS (version 20.0; SPSS Inc., Chicago, USA) and GraphPad Prism (Prism 5 for Mac OS X, Version 5.0c). The level of significance was set to *p* < 0.05 in all tests.

## Results

A three-way repeated-measures ANOVA over all pretrained groups revealed a main effect of training in the number of avoidances [*F*_(1, 178)_ = 38.39, *p* < 0.001], number of escapes [*F*_(1, 178)_ = 8.273, *p* < 0.01], number of failures [*F*_(1, 178) = 41.099_, *p* < 0.001], and escape latencies [*F*_(1, 178)_ = 41.495, *p* < 0.001]. A main effect of sex was revealed in the number of avoidances [*F*_(1, 178)_ = 13.842, *p* < 0.001], number of escapes [*F*_(1, 178)_ = 8.663, *p* < 0.01], and escape latencies [*F*_(1, 178)_ = 27.71, *p* < 0.001]. A main effect of the age at first training was revealed in the number of avoidances [*F*_(1, 178)_ = 25.787, *p* < 0.001], number of escapes [*F*_(1, 178)_ = 8.119, *p* < 0.001], number of failures [*F*_(1, 178)_ = 38.68, *p* < 0.001], and escape latencies [*F*_(1, 178)_ = 63.37, *p* < 0.001]. An effect of day of training was revealed in the number of avoidances [*F*_(4, 712)_ = 84.077, *p* < 0.001], number of escapes [*F*_(4, 712)_ = 37.092, *p* < 0.001], number of failures [*F*_(4, 712)_ = 3.939, *p* < 0.01], and escape latencies [*F*_(4, 712)_ = 24.418, *p* < 0.001].

### Sex-specific differences in avoidance learning

The repeated-measures ANOVA for the number of avoidances in the pretraining revealed a main effect of day of training [*F*_(4, 544)_ = 75.465, *p* < 0.001] and age [*F*_(4, 136)_ = 18.103, *p* < 0.001], and interactions between day of training and age [*F*_(16, 544)_ = 11.038, *p* < 0.001] and between day of training and sex [*F*_(4, 544)_ = 3.233, *p* < 0.05].

The statistical analysis for the number of escapes in the pretraining revealed a main effect of day of training [*F*_(4, 544)_ = 39.693, *p* < 0.001] and age [*F*_(4, 136)_ = 9.067, *p* < 0.001] and an interaction between day of training and age [*F*_(16, 544)_ = 11.282, *p* < 0.001].

The statistical analysis for the number of failures in the pretraining revealed a main effect of age [*F*_(4, 136)_ = 47.385, *p* < 0.001] and an interaction between day of training and age [*F*_(16, 544)_ = 3.553, *p* < 0.001].

The statistical analysis for the escape latency in the pretraining revealed a main effect of day of training [*F*_(4, 544)_ = 18.294, *p* < 0.001], age [*F*_(4, 136)_ = 72.846, *p* < 0.001], and sex [*F*_(1, 136)_ = 5.109, *p* < 0.05] and interactions between day of training and age [*F*_(16, 544)_ = 10.75, *p* < 0.001], between day of training and sex [*F*_(4, 544)_ = 4.77, *p* < 0.01] and between sex and age [*F*_(4, 136)_ = 2.718, *p* < 0.05].

The repeated-measures ANOVA for the number of avoidances in the adult training revealed a main effect of day of training [*F*_(4, 544)_ = 93.005, *p* < 0.001], age [*F*_(4, 136)_ = 18.571, *p* < 0.001], and sex [*F*_(1, 136)_ = 12.467, *p* < 0.01] and interactions between day of training and age [*F*_(16, 544)_ = 7.992, *p* < 0.001], between day of training and sex [*F*_(4, 544)_ = 2.589, *p* < 0.05], and between sex and age [*F*_(4, 136)_ = 2.886, *p* < 0.05].

The statistical analysis for the number of escapes in the adult training revealed a main effect of day of training [*F*_(4, 544)_ = 59.986, *p* < 0.001], age [*F*_(4, 136)_ = 14.41, *p* < 0.001], and sex [*F*_(1, 136)_ = 8.154, *p* < 0.01] and interactions between day of training and age [*F*_(16, 544)_ = 7.908, *p* < 0.001], between day of training and sex [*F*_(4, 544)_ = 3.27, *p* < 0.05], and between sex and age [*F*_(4, 136)_ = 3.527, *p* < 0.01].

The statistical analysis for the number of failures in the adult training revealed a main effect of day of training [*F*_(4, 544)_ = 3.836, *p* < 0.001], age [*F*_(4, 136)_ = 8.942, *p* < 0.001], and sex [*F*_(1, 136)_ = 7.663, *p* < 0.01] and interactions between day of training and age [*F*_(16, 544)_ = 1.792, *p* < 0.05] and between sex and age [*F*_(4, 136)_ = 4.159, *p* < 0.01].

The statistical analysis for the escape latency in the adult training revealed a main effect of day of training [*F*_(4, 544)_ = 14.832, *p* < 0.001], age [*F*_(4, 136)_ = 52.357, *p* < 0.001], and sex [*F*_(1, 136)_ = 55.635, *p* < 0.01] and interactions between day of training and age [*F*_(16, 544)_ = 3.999, *p* < 0.001], between day of training and sex [*F*_(4, 544)_ = 6.92, *p* < 0.001], between sex and age [*F*_(4, 136)_ = 10.423, *p* < 0.001], and between day of training, sex and age [*F*_(4, 136)_ = 3.515, *p* < 0.001].

### Ontogeny of TWA learning

**Figure 2:**
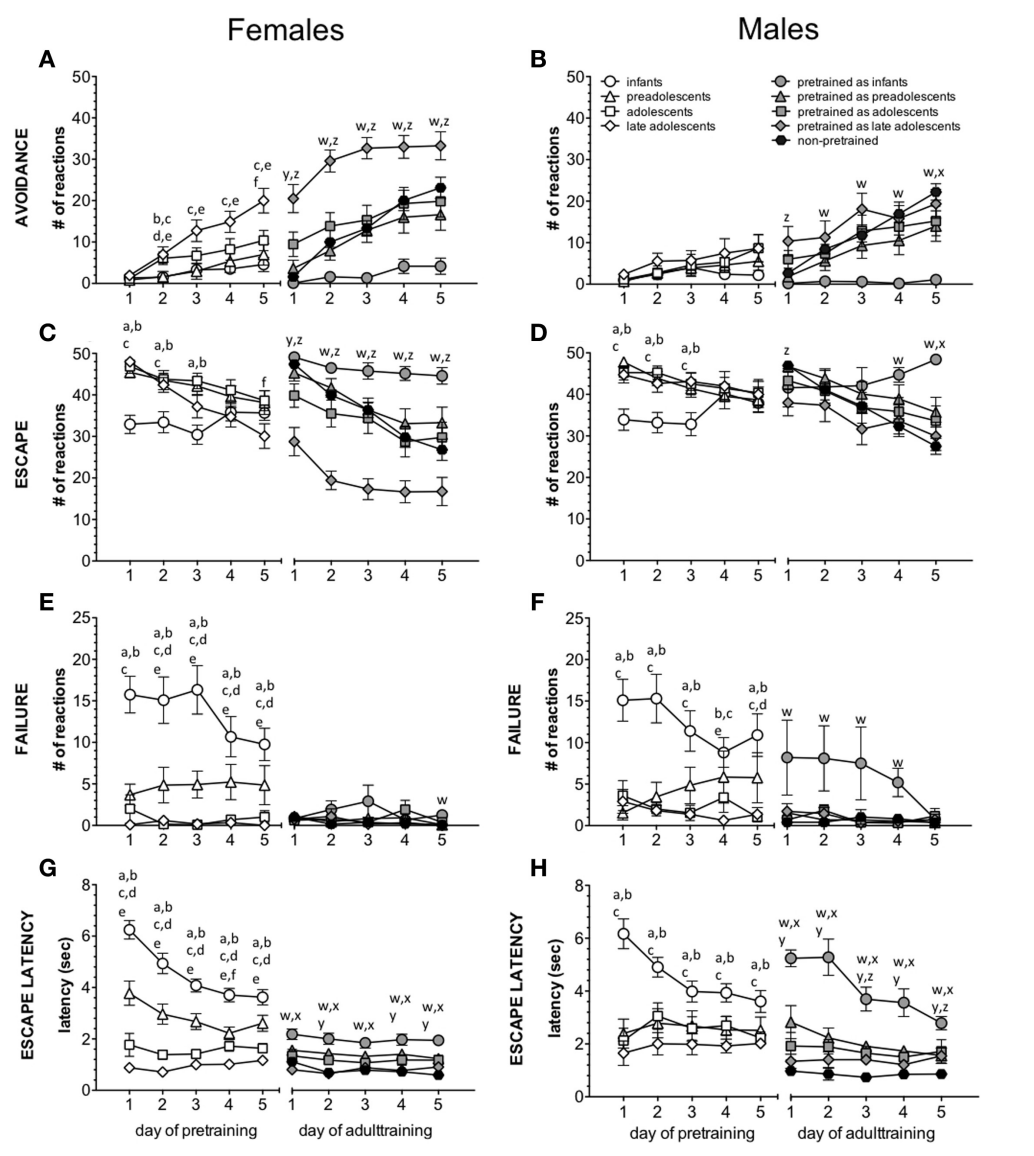
**Impact of age/age at pretraining on avoidance learning**. The left graphs show results for females, the right graphs for males. The left part of each graph summarizes the effect of age on the number of avoidances **(A,B)**, escapes **(C,D)**, and failures **(E,F)** and escape latencies **(G,H)** (mean ± s.e.m.). The letters indicate significant differences between the young age groups. The right part of each graph summarizes the effect of age at pretraining on adult active avoidance learning. The letters indicate significant differences between the pretrained groups and naive adults. a: *p* < 0.05 infants vs. preadolescents; b: *p* < 0.05 infants vs. adolescents; c: infants vs. late adolescents; d: *p* < 0.05 preadolescents vs. adolescents; e: *p* < 0.05 preadolescents vs. late adolescents; f: *p* < 0.05 adolescents vs. late adolescents; w: *p* < 0.05 pretrained as infants vs. naives; x: *p* < 0.05 pretrained as preadolescents vs. naives; y: *p* < 0.05 pretrained as adolescents vs. naives; z: *p* < 0.05 pretrained as late adolescents vs. naives.

The number of avoidance responses during pretraining is summarized in Figure 2A for females and in Figure 2B for males.

The repeated-measures ANOVA for *females* revealed a main effect of day of training [*F*_(4, 280)_ = 50.581, *p* < 0.001] and age [*F*_(4, 70)_ = 10.274, *p* < 0.001], and an interaction between day of training and age [*F*_(4, 280)_ = 5.927, *p* < 0.001], indicating a gradual increase of avoidance reactions over the 5 training days depending on age. *Post-hoc* analysis revealed that all pretraining groups, except late adolescent mice, showed significantly fewer avoidance responses compared to the adult non-pretrained animals (infants vs. adults: *p* < 0.001; preadolescents vs. adults: *p* < 0.001; adolescents vs. adults: *p* < 0.05). Infants (*p* < 0.01) and preadolescent (*p* < 0.05) animals also showed significantly fewer avoidance responses compared to the late adolescent animals. A detailed day-by-day analysis revealed an impact of age on the number of avoidance reactions on all training days, except the first day (Table 1). For the results of *post-hoc* statistics see Figure 2.

**Table 1:** **Statistical results of the day-by-day analysis obtained for the main factor age during pretraining or pretraining condition during adult training for females and males**.

	**Day of training**				
	**1st**	**2nd**	**3rd**	**4th**	**5th**
**FEMALES**					
**Pretraining**					
Avoidances	n.s.	*F*_(4, 70)_ = 7.621	*F*_(4, 70)_ = 6.499	*F*_(4, 70)_ = 8.6	*F*_(4, 70)_ = 10.318
		*p* < 0.001	*p* < 0.001	*p* < 0.001	*p* < 0.001
Escapes	*F*_(4, 70)_ = 23.94	*F*_(4, 70)_ = 4.806	*F*_(4, 70)_ = 4.883	*F*_(4, 70)_ = 3.574	*F*_(4, 70)_ = 4.38
	*p* < 0.001	*p* = 0.002	*p* = 0.002	*p* = 0.01	*p* = 0.003
Failures	*F*_(4, 70)_ = 25.557	*F*_(4, 70)_ = 21.275	*F*_(4, 70)_ = 28.17	*F*_(4, 70)_ = 12.913	*F*_(4, 70)_ = 11.447
	*p* < 0.001	*p* < 0.001	*p* < 0.001	*p* < 0.001	*p* < 0.001
Escape latencies	*F*_(4, 70)_ = 55.012	*F*_(4, 70)_ = 61.545	*F*_(4, 70)_ = 44.318	*F*_(4, 70)_ = 45.618	*F*_(4, 70)_ = 42.042
	*p* < 0.001	*p* < 0.001	*p* < 0.001	*p* < 0.001	*p* < 0.001
**Adult training**					
Avoidances	*F*_(4, 70)_ = 17.971	*F*_(4, 70)_ = 21.711	*F*_(4, 70)_ = 16.949	*F*_(4, 70)_ = 9.276	*F*_(4, 70)_ = 9.371
	*p* < 0.001	*p* < 0.001	*p* < 0.001	*p* < 0.001	*p* < 0.001
Escapes	*F*_(4, 70)_ = 17.479	*F*_(4, 70)_ = 22.332	*F*_(4, 70)_ = 13.48	*F*_(4, 70)_ = 9.709	*F*_(4, 70)_ = 8.683
	*p* < 0.001	*p* < 0.001	*p* < 0.001	*p* < 0.001	*p* < 0.001
Failures	n.s.	n.s.	n.s.	n.s.	*F*_(4, 70)_ = 3.669
					*p* = 0.009
Escape latencies	*F*_(4, 70)_ = 9.213	*F*_(4, 70)_ = 18.789	*F*_(4, 70)_ = 6.929	*F*_(4, 70)_ = 12.686	*F*_(4, 70)_ = 17.756
	*p* < 0.001	*p* < 0.001	*p* < 0.001	*p* < 0.001	*p* < 0.001
**MALES**					
**Pretraining**					
Avoidances	*F*_(4, 66)_ = 2.849	*F*_(4, 66)_ = 4.466	*F*_(4, 66)_ = 3.763	*F*_(4, 66)_ = 7.744	*F*_(4, 66)_ = 11.969
	*p* = 0.031	*p* = 0.003	*p* = 0.008	*p* < 0.001	*p* < 0.001
Escapes	*F*_(4, 66)_ = 13.484	*F*_(4, 66)_ = 6.047	*F*_(4, 66)_ = 3.242	*F*_(4, 66)_ = 3.428	*F*_(4, 66)_ = 5.646
	*p* < 0.001	*p* < 0.001	*p* = 0.017	*p* = 0.013	*p* = 0.001
Failures	*F*_(4, 66)_ = 18.58	*F*_(4, 66)_ = 18.982	*F*_(4, 66)_ = 7.825	*F*_(4, 66)_ = 3.987	*F*_(4, 66)_ = 7.465
	*p* < 0.001	*p* < 0.001	*p* < 0.001	*p* = 0.006	*p* < 0.001
Escape latencies	*F*_(4, 66)_ = 24.172	*F*_(4, 66)_ = 14.38	*F*_(4, 66)_ = 12.629	*F*_(4, 66)_ = 15.843	*F*_(4, 66)_ = 14.24
	*p* < 0.001	*p* < 0.001	*p* < 0.001	*p* < 0.001	*p* < 0.001
**Adult training**					
Avoidances	*F*_(4, 66)_ = 3.807	*F*_(4, 66)_ = 2.957	*F*_(4, 66)_ = 4.231	*F*_(4, 66)_ = 4.686	*F*_(4, 66)_ = 7.187
	*p* = 0.008	*p* = 0.026	*p* = 0.004	*p* = 0.002	*p* < 0.001
Escapes	*F*_(4, 66)_ = 3.271	n.s.	n.s.	*F*_(4, 66)_ = 2.877	*F*_(4, 66)_ = 7.262
	*p* = 0.017			*p* = 0.029	*p* < 0.001
Failures	*F*_(4, 66)_ = 3.9	*F*_(4, 66)_ = 4.765	*F*_(4, 66)_ = 3.04	*F*_(4, 66)_ = 6.218	n.s.
	*p* = 0.007	*p* = 0.002	*p* = 0.023	*p* < 0.001	
Escape latencies	*F*_(4, 66)_ = 19.334	*F*_(4, 66)_ = 20.698	*F*_(4, 66)_ = 25.79	*F*_(4, 66)_ = 22.634	*F*_(4, 66)_ = 8.267
	*p* < 0.001	*p* < 0.001	*p* < 0.001	*p* < 0.001	*p* < 0.001

The statistical analysis for *males* revealed a main effect of day of training [*F*_(4, 264)_ = 26.541, *p* < 0.001] and age [*F*_(4, 66)_ = 8.683, *p* < 0.001] and an interaction between day of training and age [*F*_(16, 264)_ = 6.41, *p* < 0.001], indicating a gradual increase of avoidance reactions over the 5 training days depending on age. *Post-hoc* test revealed that all pretraining groups showed significantly fewer avoidance reactions compared to the adult non-pretrained mice (infants vs. adults: *p* < 0.001; preadolescents vs. adults: *p* < 0.001; adolescents vs. adults: *p* < 0.001; late adolescents vs. adults: *p* < 0.01). A detailed day-by-day analysis revealed an impact of age on avoidance performance on all training days (Table 1). For the results of *post-hoc* statistics see Figure 2.

The number of escapes during pretraining is summarized in Figure 2C for females and in Figure 2D for males.

The statistical analysis for *females* revealed a main effect of day of training [*F*_(4, 280)_ = 27.537, *p* < 0.001] and age [*F*_(4, 70)_ = 5.293, *p* = 0.001] and an interaction between day of training and age [*F*_(16, 280)_ = 5.761, *p* < 0.001], indicating a gradual decrease in the number of escapes over the 5 training days depending on age. *Post-hoc* analysis revealed that preadolescent (*p* < 0.01) and adolescent (*p* < 0.01) mice showed significantly more escapes compared to adult non-pretrained animals. The infants displayed significantly fewer escapes compared to preadolescent (*p* < 0.01) and adolescent (*p* < 0.001) animals. A detailed day-by-day analysis revealed an impact of age on escape reactions on all training days (Table 1). For the results of *post-hoc* statistics see Figure 2.

The statistical analysis for *males* revealed a main effect of day of training [*F*_(4, 264)_ = 13.357, *p* < 0.001] and age [*F*_(4, 66)_ = 4.182, *p* < 0.05] and an interaction between day of training and age [*F*_(16, 264)_ = 6.836, *p* < 0.001], indicating a gradual decrease in the number of escapes over the 5 training days depending on age. *Post-hoc* test revealed that preadolescent (*p* < 0.05), adolescent (*p* < 0.01), and late adolescent (*p* < 0.05) mice showed significantly more escapes compared to adult non-pretrained animals. The infants displayed significantly fewer escapes compared to preadolescent (*p* < 0.05), adolescent (*p* < 0.01), and late adolescent (*p* < 0.05) animals. A detailed day-by-day analysis revealed an effect of age on escape reactions on all training days (Table 1). For the results of *post-hoc* statistics see Figure 2.

The number of failures during pretraining is summarized in Figure 2E for females and in Figure 2F for males.

The statistical analysis for *females* revealed a main effect of age [*F*_(4, 70)_ = 41.635, *p* < 0.001] and an interaction between day of training and age [*F*_(16, 280)_ = 1.802, *p* < 0.05], indicating a gradual decrease in the number of failures over the 5 training days depending on age. *Post-hoc* analysis revealed that infant (*p* < 0.001) and preadolescent (*p* < 0.001) animals showed significantly more failures compared to adult non-pretrained animals. The infant animals showed also significantly more failures than preadolescent (*p* < 0.001), adolescent (*p* < 0.001), and late adolescent (*p* < 0.001) animals and the preadolescent animals displayed significantly more failures than adolescent (*p* < 0.01) and late adolescent (*p* < 0.01) mice. A detailed day-by-day analysis revealed an impact of age on failures on all training days (Table 1). For the results of *post-hoc* statistics see Figure 2.

The statistical analysis for *males* revealed a main effect of age [*F*_(4, 66)_ = 14.403, *p* < 0.001] and an interaction between day of training and age [*F*_(16, 264)_ = 2.75, *p* < 0.001], indicating a gradual decrease in the number of failures over the 5 training days depending on age. *Post-hoc* analysis revealed that infant (*p* < 0.001) and preadolescent (*p* < 0.05) animals showed significantly more failures compared to adult non-pretrained animals. The infant animals showed also significantly more failures than preadolescent (*p* < 0.001), adolescent (*p* < 0.001), and late adolescent (*p* < 0.001) animals. A detailed day-by-day analysis revealed an impact of age on failures on all training days (Table 1). For the results of *post-hoc* statistics see Figure 2.

The escape latency during infant/adolescent training is summarized in Figure 2G for females and in Figure 2H for males.

The statistical analysis for *females* revealed a main effect of day of training [*F*_(4, 280)_ = 18.854, *p* < 0.001] and age [*F*_(4, 70)_ = 102.906, *p* < 0.001] and an interaction between day of training and age [*F*_(16, 280)_ = 6.536, *p* < 0.001], indicating a gradual decrease in the escape latency over the 5 training days depending on age. *Post-hoc* test revealed longer escape latencies of infants compared to preadolescent (*p* < 0.001), adolescent (*p* < 0.001) and late adolescent (*p* < 0.001) mice as well as adult non-pretrained (*p* < 0.001) mice. The preadolescent animals showed significantly longer escape latencies compared to the adolescent (*p* < 0.001) and late adolescent (*p* < 0.001) as well as adult non-pretrained (*p* < 0.001) animals. The adolescent mice showed significantly longer escape latencies compared to late adolescent (*p* < 0.01) and adult non-pretrained (*p* < 0.001) animals. A detailed day-by-day analysis revealed an impact of age on escape latencies on all training days (Table 1). For the results of *post-hoc* statistics see Figure 2.

The statistical analysis for *males* revealed a main effect of day of training [*F*_(4, 264)_ = 5.124, *p* < 0.01] and age [*F*_(4, 66)_ = 20.192, *p* < 0.001] and an interaction between day of training and age [*F*_(16, 264)_ = 5.655, *p* < 0001], indicating a gradual decrease in the escape latency over the 5 training days depending on age. *Post-hoc* test revealed longer escape latencies of infants compared to preadolescent (*p* < 0.001), adolescent (*p* < 0.001), and late adolescent (*p* < 0.001) mice as well as adult non-pretrained (*p* < 0.001) mice. The adult non-pretrained animals showed significantly shorter escape latencies compared to preadolescent (*p* < 0.001), adolescent (*p* < 0.001), and late adolescent (*p* < 0.05) animals. A detailed day-by-day analysis revealed an impact of age on escape latencies on all training days (Table 1). For the results of *post-hoc* statistics see Figure 2.

### Impact of TWA pretraining on adult avoidance learning

The number of avoidances during adult training is summarized in Figure 2A for females and in Figure 2B for males.

The statistical analysis for *females* revealed a main effect of day of training [*F*_(4, 280)_ = 53.765, *p* < 0.001] and age [*F*_(4, 70)_ = 16.543, *p* < 0.001] and an interaction between day of training and age [*F*_(16, 280)_ = 3.848, *p* < 0.001], indicating a gradual increase of avoidance reactions over the 5 training days depending on the age at pretraining. *Post-hoc* test revealed that mice pretrained as infants showed significantly fewer avoidance reactions compared to animals pretrained in preadolescence (*p* < 0.01), adolescence (*p* < 0.001), and late adolescence (*p* < 0.001) as well as adult non-pretrained (*p* < 0.001) mice. In contrast, females pretrained in late adolescence displayed significantly more avoidances compared to animals pretrained in preadolescence (*p* < 0.001) and adolescence (*p* < 0.001) as well as adult non-pretrained (*p* < 0.001) animals. A detailed day-by-day analysis revealed an impact of age at pretraining on avoidance reactions on all training days (Table 1). For the results of *post-hoc* statistics see Figure 2.

The statistical analysis for *males* revealed a main effect of day of training [*F*_(4, 264)_ = 39.407, *p* < 0.001] and age [*F*_(4, 66)_ = 5.169, *p* = 0.001] and an interaction between day of training and age [*F*_(16, 264)_ = 3.72, *p* < 0.001], indicating a gradual increase of avoidance reactions over the 5 training days depending on the age at pretraining. *Post-hoc* test revealed that mice pretrained as infants showed significantly fewer avoidances compared to animals pretrained in preadolescence (*p* < 0.05), adolescence (*p* < 0.01) and late adolescence (*p* < 0.001) as well as adult non-pretrained (*p* < 0.001) mice. A detailed day-by-day analysis revealed an impact of age at pretraining on avoidance reactions on all training days (Table 1). For the results of *post-hoc* statistics see Figure 2.

The number of escapes during adult training is summarized in Figure 2C for females and in Figure 2D for males.

The statistical analysis for *females* revealed a main effect of day of training [*F*_(4, 280)_ = 45.854, *p* < 0.001] and age [*F*_(4, 70)_ = 16.447, *p* < 0.001] and an interaction between day of training and age [*F*_(16, 280)_ = 3.27, *p* < 0.001], indicating a gradual decrease in the number of escapes over the 5 training days depending on the age at pretraining. *Post-hoc* test revealed that mice pretrained as infants showed significantly more escape reactions compared to animals pretrained in preadolescence (*p* < 0.05), adolescence (*p* < 0.001), and late adolescence (*p* < 0.001) as well as adult non-pretrained (*p* < 0.01) animals. In contrast, females pretrained in late adolescence displayed significantly fewer escape reactions compared to animals pretrained in preadolescence (*p* < 0.001) and adolescence (*p* < 0.001) as well as adult non-pretrained (*p* < 0.001) animals. A detailed day-by-day analysis revealed an impact of age at pretraining on escape reactions on all training days (Table 1). For the results of *post-hoc* statistics see Figure 2.

The statistical analysis for *males* revealed a main effect of day of training [*F*_(4, 264)_ = 18.919, *p* < 0.001] and an interaction between day of training and age [*F*_(16, 264)_ = 5.215, *p* < 0.001], indicating a gradual decrease in the number of escapes over the 5 training days depending on the age at pretraining. *Post-hoc* test revealed that mice pretrained as infants showed significantly more escape reactions compared to mice pretrained in late (*p* < 0.01) adolescence and to non-pretrained (*p* < 0.05) adults. Males pretrained in late adolescence displayed fewer escapes than mice pretrained in preadolescence (*p* < 0.05). A detailed day-by-day analysis revealed an impact of age at pretraining on escape reactions on 1st, 4th, and 5th training day (Table 1). For the results of *post-hoc* statistics see Figure 2.

The number of failures during adult training is summarized in Figure 2E for females and in Figure 2F for males.

The statistical analysis for *females* revealed a main effect of age [*F*_(4, 70)_ = 2.765, *p* < 0.05]. The *post-hoc* test revealed that mice pretrained in infancy showed significantly more failures compared to females pretrained in preadolescence (*p* < 0.05) or late adolescence (*p* < 0.05) and to non-pretrained (*p* < 0.01) adults. A detailed day-by-day analysis revealed an impact of age at pretraining on failures only on the last training day (Table 1). For the results of *post-hoc* statistics see Figure 2.

The statistical analysis for *males* revealed a main effect of day of training [*F*_(4, 264)_ = 2.949, *p* < 0.05] and age [*F*_(4, 66)_ = 6.338 *p* < 0.001] and an interaction between day of training and age [*F*_(16, 264)_ = 1.713, *p* < 0.05], indicating a gradual decrease in the number of failures over the 5 training days depending on the age at pretraining. *Post-hoc* test revealed that males pretrained as infants showed significantly more failures compared to animals pretrained in preadolescence (*p* < 0.001), adolescence (*p* < 0.001), and late adolescence (*p* < 0.001) as well as adult non-pretrained (*p* < 0.001) animals. A detailed day-by-day analysis revealed an impact of age at pretraining on failures from 1st to 4th training day (Table 1). For the results of *post-hoc* statistics see Figure 2.

The escape latency during adult training is summarized in Figure 2G for females and in Figure 2H for males.

The statistical analysis for *females* revealed a main effect of day of training [*F*_(4, 280)_ = 3.019, *p* < 0.05] and age [*F*_(4, 70)_ = 16.447, *p* < 0.001]. *Post-hoc* test revealed that mice pretrained as infants showed significantly longer escape latencies compared to mice pretrained in preadolescence (*p* < 0.001), adolescence (*p* < 0.001), and late adolescence (*p* < 0.001) as well as adult non-pretrained (*p* < 0.001) animals. Females pretrained in preadolescence displayed significantly longer escape latencies compared to animals pretrained in late adolescence (*p* < 0.001) and to non-pretrained adults (*p* < 0.001). Also females pretrained in adolescence showed significantly longer escape latencies compared to animals pretrained in late adolescence (*p* < 0.05) and to non-pretrained adults (*p* < 0.01). A detailed day-by-day analysis revealed an impact of age at pretraining on escape latencies on all training days (Table 1). For the results of *post-hoc* statistics see Figure 2.

The statistical analysis for *males* revealed a main effect of day of training [*F*_(4, 264)_ = 13.07, *p* < 0.001] and age [*F*_(4, 66)_ = 29.913, *p* < 0.001] and an interaction between day of training and age [*F*_(16, 264)_ = 4.464, *p* < 0.01], indicating a gradual decrease of escape latency over the 5 training days depending on the age at pretraining. The *post-hoc* test revealed that mice pretrained as infants showed significantly longer escape latencies compared to animals pretrained in preadolescence (*p* < 0.001), adolescence (*p* < 0.001), and late adolescence (*p* < 0.001) as well as adult non-pretrained (*p* < 0.001) animals. Males pretrained in preadolescence displayed longer escape latencies compared to mice pretrained in late adolescence (*p* < 0.05) and compared to adult non-pretrained (*p* < 0.001) males. Animals pretrained in adolescence showed longer escape latencies compared to adult non-pretrained (*p* < 0.01) mice. A detailed day-by-day analysis revealed an impact of age at pretraining on escape latencies on all training days (Table 1). For the results of *post-hoc* statistics see Figure 2.

## Discussion

### Ontogeny of TWA learning in mice

Emotional and cognitive experience in childhood affects the development of neural networks, behavioral profiles as well as the capacity of learning and memory. The behavioral as well as the brain structural changes critically depend on the developmental time period in which the experience is encountered and the memory persistence is proportional to the age at the time of training (Zaharia et al., 1996; Heim and Nemeroff, 2001; Pollak, 2003; Romeo et al., 2003; Bock et al., 2005; Ruedi-Bettschen et al., 2005). With respect to the ontogeny of TWA learning the impact of different parameters (e.g., sex, type of CS, UCS intensity) was studied in rats and revealed a correlation between learning performance and age (Bauer, 1978, 1982). Most studies, which investigated the ontogeny of learning and memory, were conducted in rats (e.g., McLaughlin et al., 1975; McNamara et al., 1977; Bauer, 1978; Myslivecek and Hassmannova, 1979; Rudy et al., 1987), whereas less experimental data are available for mice (Hefner and Holmes, 2007; Ito et al., 2009; Akers et al., 2012). Due to the increasing use of transgenic and knock-out mice as model organisms in research and since behavioral traits cannot be simply generalized or extrapolated between rats and mice (Whishaw et al., 2001), there is an increasing necessity to characterize mouse cognitive behavior in more detail, in particular in relation to developmental traits. The present study revealed for both male and female mice that TWA learning is age-dependent and that females (but not males) trained during late-adolescence perform better than the infant and preadolescent group. These findings are in line with results from studies in rats which report an increase of TWA learning during development (Bauer, 1978; Myslivecek and Hassmannova, 1979; Schäble et al., 2007; Gruss et al., 2010).

An animal usually learns associations by trial and error, which requires not only a strong primary incentive, but also involves reinforcement feedback to its own behavioral responses as motivator to continuously focus on this task and thereby optimize its behavioral strategies (Scheich and Brosch, 2013). What are the constraints that determine the deficits in active avoidance learning observed in infant mice compared to the performance of adolescent and adult animals? There are two basic, functionally linked concepts, “immature brain” theories and “ongoing brain maturation” theories (Josselyn and Frankland, 2012), which address this question. Key structures for memory formation and storage, e.g., cortex and hippocampus with their protracted postnatal development and/or sensory and motor cortical functions, are still immature at the time of TWA training. In other words, the neuronal and molecular events underlying ongoing brain maturation at a given developmental phase limit learning and memory functions. For instance, motor pathways mediating motor activity, which is a critical prerequisite to perform the TWA task, might be too immature and thereby limit the motor skills of the animal. However, this appears to be unlikely since there is evidence that infant mice have no major sensory or motor impairments, i.e., they are capable to change between the shuttle box compartments. Several studies (Williams and Scott, 1953; Fox, 1965; van Abeelen and Schoones, 1977) have shown that responses to most reflexes are adult-like between PND 13 and 16. Moreover, their motor skills have been documented in experiments, which show that eleven-day-old mice learn an escape response in a shock-escape T-maze, where the mice have to run to the goal arm (Nagy and Murphy, 1974).

Prefronto-limbic circuits, i.e., prefrontal cortical regions, the amygdala, and the hippocampal formation, which are critically involved in this learning task (Molino, 1975; Brennan et al., 1977; Gray and McNaughton, 1983; Lacroix et al., 1998; Stark et al., 2000; Choi et al., 2010), are still immature and developing in infancy and adolescence (Spear, 2000, 2004) and thus might be “overburdened” by the complexity of the TWA task. It is important to emphasize that, in contrast to other aversive learning paradigms such as fear conditioning, TWA learning is highly complex as it involves an initial phase of fear conditioning followed by a conditioned escape response, which is then optimized and transferred into an avoidance response. Our experiments clearly demonstrate that mice rapidly learn an escape response (reflected in a decrease of escape latencies and failures), indicating that they have learned that changing the compartment terminates the punishing signal. However, the main constraint in the infant mice might be their inability to transfer the rather stereotype escape responses into an avoidance strategy, as it is typically observed in late adolescent and adult mice. Learning an active avoidance strategy requires the incorporation of the prediction error into the learning process (Scheich and Brosch, 2013). The consequence of understanding the association between the CS (tone) and the UCS (footshock) is that the animal predicts or expects the UCS when it hears the CS. However, as there is no footshock whenever the animal—initially more or less randomized—changes the compartment after the onset of the CS and *prior to* the onset of the UCS the animal eventually realizes that it cannot only escape from but also to avoid the unpleasant UCS (prediction error). In view of the strikingly opposing consequences of infant and adolescent TWA training on adult learning the question arises, whether and in which way the memory differs in relation to the age during pretraining. As a measure of learning success we observed that subtracting the number of (initially more or less “random”) avoidances on the first training day from the number of (goal-directed) avoidances during the fifth training day (Hefner and Holmes, 2007) revealed significant differences only in the preadolescent, adolescent and late adolescent groups as well as in the non-pretrained adults, but not in the infant group (data not shown). This indicates that their chance of experiencing a prediction error is too low to develop a successful avoidance strategy.

In addition to these cognitive restraints, the emotional challenge caused by the conflict situation, i.e., to return to the compartment, where the animal has been previously punished, might be too difficult to cope with for infant mice, due to the immaturity and ongoing maturation of emotional, in particular anxiety-related pathways.

### Age-dependent consequences of TWA pretraining on adult avoidance learning

In the present study we explored the long-term consequences of learning during infancy or adolescence on adult learning performance. As already mentioned, infancy and adolescence are time windows, which are characterized by pronounced functional maturation of neural circuits (review Spear, 2000). On the neuronal level there is a host of evidence that positive as well as adverse environmental conditions and experience during these critical time windows of neuronal development and synaptic reorganization dramatically interfere with the maturation of functional brain pathways (Rice and Barone, 2000; Chambers et al., 2003; Bock et al., 2005, 2013; Sullivan et al., 2006; Hunt et al., 2007; Lupien et al., 2009; Flanigan and Cook, 2011). On the behavioral level a variety of studies in humans and animals revealed that manipulations in early life such as handling, maternal separation and footshock either enhanced (Domes et al., 2002; Smeets et al., 2007; Schwabe et al., 2008) or impaired (Kirschbaum et al., 1996; Elzinga et al., 2005; Diamond et al., 2006) learning and memory functions depending on the timing of the manipulation, sex and the task (for review see Kosten et al., 2012). For example, juvenile rodents, which were exposed to different types of stressors during prepuberty, showed impaired avoidance learning in adulthood (Tsoory and Richter-Levin, 2006; Peleg-Raibstein and Feldon, 2011). On the other hand, in contrast to our experiments, which showed impaired active avoidance learning after exposure to CS paired with *escapable* footshock during infancy, mice exposed to different intensities of *inescapable* footshocks without paired CS during infancy (PND 15-20) displayed better avoidance learning in adulthood (Denenberg and Karas, 1959; Denenberg and Bell, 1960; Bell and Denenberg, 1963).

Our results show for the first time that the impact of pretraining on adult avoidance learning critically depends on the age of the animal during exposure to the TWA training. Pretraining in infants (starting on PND 17) results in a dramatic impairment of adult avoidance learning, i.e., they need more time to change the compartment and to locate the exit to the other compartment compared to non-pretrained adults. This negative outcome of infant TWA training is in striking contrast to findings of similar experiments in rats (pretrained at the same age as our infant mice), which displayed accelerated and improved avoidance learning in adulthood (Schäble et al., 2007; Gruss et al., 2010). The discrepant outcome of infant TWA training may at least in part be due to the less complex synaptic organization in the mouse brain compared to rats, which might not only restrain behavioral flexibility but also limit the potential for learning- and memory-induced synaptic plasticity in the mouse brain (Pellis and Pellis, 1998; Pellis and Iwaniuk, 2000; Whishaw et al., 2001). Furthermore, the high amount of “punishment” (as reflected by very long escape latencies and a high number of failures during the infant TWA training), which in particular is experienced in the infant group during TWA training, may induce strong memories of the aversive components, i.e., the UCS and the context (shuttle box). This may induce elevated general anxiety, which is remembered during adult retraining and these animals might be “overwhelmed” by fear when placed in the shuttle-box, which impairs avoidance learning in adulthood. Experiments with adult male mice showed that preexposure to only UCS induces an attenuation of the TWA learning (Chang et al., 2007) and can lead to a kind of learned helplessness (Overmier and Seligman, 1967). Alternatively, the escape strategy learned in infancy might become “imprinted” into their brain, and this “hard-wired” behavior may limit their behavioral flexibility including the switch between escape and avoidance responses later in life.

In contrast to the impaired avoidance learning observed in mice pretrained during infancy, mice pretrained in late-adolescence showed significantly more avoidance responses on the first adult training day compared to non-pretrained adults. These results indicate that the animals remember the avoidance strategy, which they have started to learn during adolescent pretraining and incorporate this memory into the still ongoing learning process as adults and thereby optimize the avoidance strategy. This is in line with findings that indicate that late adolescence appears to be the optimal age for avoidance learning in mice (Stavnes and Sprott, 1975), as the maturation of most learning and memory-relevant circuits is completed (Altman and Bayer, 1975; Stanton, 2000; Esposito et al., 2005).

With respect to long-term memory functions it is remarkable that the delay between the infant/adolescent pretraining and adult training was several weeks, i.e., much longer than the 24 or 48 h intervals which are commonly used for testing long-term memory (e.g., Gerlai, 1998; Bolivar et al., 2001; Chauveau et al., 2009; Huang et al., 2011). Only few studies using the contextual fear conditioning paradigm retested the animals after several weeks (Balogh and Wehner, 2003; Akers et al., 2012). For instance, adult C57Bl/6 mice showed a high level of freezing upon context and only CS (tone) even 60 days after conditioning (Balogh and Wehner, 2003), whereas infant mice appear not to remember a context-shock association after 7 or more days, and adolescent and adult mice can remember at least 28 days (Akers et al., 2012). In the present study adult avoidance learning was performed at the same time point (PND 80-84) for all adult pretraining groups. It is important to point out that the delay between pretraining and adult retraining in the animals pretrained in late adolescence was shorter (6 weeks) than in the mice pretrained in infancy (9 weeks). However, an effect of this temporal delay on long-term memory appears unlikely since mice pretrained during late adolescence and retrained after 9 weeks as adults displayed the same increase in the number of avoidances as the animals retrained after 6 weeks (data not shown). Thus, the improvement of adult learning performance in the pretrained late adolescent group is more likely due to better long-term memory storage rather than to the shorter temporal delay used in the present study.

### Sex-specific differences in avoidance learning

Female rodents are often excluded from behavioral experiments due to the assumption that the estrous cycle affects behavior, in particular learning performance, and thus should result in a higher variability of their behavioral performance (Farr et al., 1995). For instance, studies using a (different from the one used in our study) footshock avoidance paradigm reported that male CD-1 mice learn faster and show lower performance variability than females (Farr et al., 1995). This is in contrast to findings of the present and previous studies (Schäble et al., 2007; Gruss et al., 2010), where on average a more homogeneous TWA learning was observed in adult female animals compared to males. A meta-analysis of sex differences in learning and memory functions revealed that sex differences cannot be generalized as they also dependent on a variety of parameters including the rodent strain, age, learning task, and the design of the experimental protocol (Farr et al., 1995; Frick et al., 2000; Jonasson, 2005).

Our present experiments revealed a clear sex difference in the group of late-adolescent mice. It is known, that sex differences in behavior often develop during or after adolescence (Krasnoff and Weston, 1976; Kanit et al., 2000; Hodes and Shors, 2005). Adolescence is not only characterized by changes in hormone concentration and brain anatomical structure (Gorski et al., 1978; Diamond et al., 1983; Matsumoto, 1991), but also by remodeling of cortical and limbic circuits, which are essentially involved in behavioral functions (Palanza, 2001; Sisk and Zehr, 2005). Several studies using a variety of paradigms revealed that males and females display little or no behavioral differences prior to puberty, whereas in adulthood the genders react differently. For instance, while adult females outperformed males in trace eyeblink conditioning, no sex differences were observed before and during puberty (Hodes and Shors, 2005). This is in line with our observations, that female mice showed better avoidance learning during late adolescence compared to males, and the beneficial impact of late-adolescent pretraining on adult learning is more pronounced in females compared to males.

Taken together, we show here for the first time that the beneficial or detrimental outcome of pretraining in childhood and prepuberty strongly depends on the age during which TWA training is encountered, indicating that different, age-dependent “memory traces” might be formed, which are recruited during adult TWA training, and thereby either facilitate or impair adult learning.

### Conflict of interest statement

The authors declare that the research was conducted in the absence of any commercial or financial relationships that could be construed as a potential conflict of interest.
